# Socio-cultural context of eating disorders in Poland

**DOI:** 10.1186/s40337-016-0093-3

**Published:** 2016-03-18

**Authors:** Maciej Wojciech Pilecki, Kinga Sałapa, Barbara Józefik

**Affiliations:** Department of Child and Adolescent Psychiatry, Jagiellonian University Medical College, ul. Kopernika 21a, 31-501 Kraków, Poland; Department of Bioinformatics and Telemedicine, Jagiellonian University Medical College, ul. Św. Łazarza 16, 31-530 Kraków, Poland; Department of Child and Adolescent Psychiatry, Laboratory of Psychology and Systemic Psychotherapy, Jagiellonian University Medical College, ul. Kopernika 21a, 31-501 Kraków, Poland

**Keywords:** Anorexia nervosa, Bulimia nervosa, Westernisation, Culture

## Abstract

**Background:**

The goal of this study was to assess the relationship between sociocultural factors and clinical eating disorders during the intensive process of Westernisation in Poland that occurred after 1989. The study population included girls diagnosed with an eating disorder according to DSM-IV criteria (*n* = 47 anorexia nervosa restrictive type [ANR], *n* = 16 anorexia binge/purge type [ANBP], *n* = 34 bulimia nervosa [BN], *n* = 19 eating disorder not otherwise specified [EDNOS]) who received consultation for the first time between 2002 and 2004 in the Department of Clinical Child and Adolescent Psychiatry, University Hospital, Kraków, Poland. The study included an age-matched normal control group [NOR] of 85 schoolgirls from Kraków.

**Methods:**

Relationships between two given qualitative features were investigated using the chi-square test or Fisher’s exact test. Correspondence analysis was applied to graphically explore the relationship. The Kruskal-Wallis test with the Bonferroni was performed to compare quantitative results across groups.

**Results:**

Objective sociodemographic variables and responses to the 62-item Questionnaire of Socio-cultural Context were measured. The mothers of ANBP and BN patients were less professionally active than mothers of ANR patients and NOR subjects. Subjective socio-cultural factors were more relevant for the BN group than the ANR group. Questionnaire responses in the ANBP group were more similar to those in the BN group than to those in the ANR group. The most unambiguous and specific characteristic of the ANR group was a sense of belonging to the middle class. Variables that differentiated the BN group from the NOR group included the importance attached to thinness treated as an expression of power and control over one’s self, as well as a multifaceted negative evaluation of one’s own family, including a negative assessment of the position of women and parental lack of concern for appearance and principles of nutrition. All patients, regardless of diagnosis, identified with other people with similar problems and considered anorexia and bulimia to be a major issue of their generation and social environment.

**Conclusions:**

The results of this first in Poland exploratory study of socio-cultural context of eating disorders indicate the importance of both objective and subjective socio-cultural factors in eating disorders in the group studied.

## Background

Socio-cultural context is acknowledged as an important risk factor for the development and specificity of clinical eating disorders [EDs] [1, 2].

Although EDs are not exclusive to contemporary Western culture, process of Westernisation in Japan after the Second World War and in countries of the former Eastern Bloc after the lifting of the Iron Curtain is accompanied by the increase of EDs prevalence in this countries [3, 4]. The same applies for the process of acculturation and over-identification to the occurrence of EDs after immigration to countries of Western culture [5] or changes in race identity within the sphere of Western countries [6, 7]. In this context, EDs are sometimes referred to as culture-bound syndromes [8].

Over the years, the dominant view has been that the highest risk of EDs occurs among girls that attend private schools in large cities and that come from families who place a high value on achievement and adaptability [9, 10]. Currently, however, it is difficult to discern a clear relationship between the occurrence of EDs and the social class of patients, and studies of different populations (e.g. clinical, community) contradict each other [11]. This may be due to recent expansion of the disease across different social strata [12], or to erroneous conclusions in the initial stages of research that relied on the analysis of data collected from private clinics, which would have been attended by only rich patients [4, 13]. Social class may be significant not only as a risk factor, but also as a factor in the course of EDs [14].

More conclusive evidence of the importance of cultural context in EDs is provided by research that has looked for mechanisms linking general characteristics of Western culture with the risk of EDs occurring in specific individuals. Socio-cultural pressure to be thin is a relevant risk factor for EDs that acts on an individual level [15]. Experimental findings have confirmed that the perceived pressure to be thin and the internalisation of the thin ideal is a causal risk factor for body dissatisfaction, dieting, negative affect and eating pathology [16, 17]. Body preoccupation and the fear of weight gain correspond with the above phenomena and are also a significant risk factor for eating problems [17, 18].

Although the importance of thin ideal can be observed in different cultures, it is notably associated with Western culture values [19]. It may be responsible for dieting, even in the absence of body dissatisfaction, as the result of a desire to adapt to applicable standards [15]. Researchers suggest that the construction of an ideal body has become mandatory for modern women and cause a deep sense of imperfections of one’s own body and a sense of guilt and shame [20–22]. From this perspective, thinness may be regarded as a body modification specific to Western culture [23, 24].

Mass media, family communication and the peer group all have an important impact on the construction of the sociocultural pressure to be thin [25–29]. Many studies have shown that mass media is an important element influencing body dissatisfaction [30], or at least awareness and attention to one’s body [31, 32]. However, assigning a significant role to media exposure may provide too narrow a view of the problem. Mass media can exert influence through complementary stimuli, for example, the confrontation of one’s own body with the bodies of models seen in advertisements, participation in the lives of celebrities through social media and celebrity gossip portals, coveting things advertised by models.

EDs have become a separate aspect of mass culture. They have gained independent, culture-forming significance as Internet phenomena such as *Thinspiration*, *Pro-ana* and *Mia* [33–35]. One may wonder about the importance of cultural factors as risk factors, or at the very least maintenance factors, in the development of AN and BN.

Some research confirms the importance parental attitudes towards body standards and weight loss on the development of an eating pathology [27, 36], especially the critical comments of loved ones on body shape, weight and food [37]. However, there is an absence of convincing evidence that this dependency, which has been confirmed in the general population, is significant as a risk factor for EDs [25, 29]. On the other hand, EDs in mothers do constitute an important factor for development of eating pathology in daughter [38].

Persons who constitute a peer group frequently have similar problems associated with their relationship to their bodies and their dietary behaviour [39] Peer group can have a significant impact on body image, dieting attitudes and eating pathology, especially among individuals with BN [29], and that the influence of peers is one of the strongest cultural factors correlated with body dissatisfaction [28].

Despite some doubts about the importance of culture in EDs, some of its aspects appear to be an important risk factor [40]. However, it should be noted that the majority of convincing empirical studies address socio-cultural risk factors for binge eating and symptoms of bulimia or general symptoms of eating pathology, rather than symptoms of anorexia [15]. The importance of cultural factors in the development of EDs has been confirmed in prospective studies [41], and indirect confirmation has been provided by the development of effective prevention programs [42]. However, it is not clear why, despite much evidence supporting the significance of socio-cultural pressure on women to be thin in the aetiology of EDs, relatively few women living in contemporary Western cultures are affected by EDs [43].

Countries in the former Eastern Bloc, such as Poland, pose an interesting problem for the role of sociocultural context in development of EDs. Clinical cases of eating disorders prior to political change of 1989 were rare. Prevalence of eating disorders in general population was not studied, or methodology of this studies is unknown or unreliable [4, 44]. After 1989 EDs have become one of the most common mental disorders faced by young girls in Poland [45]. In Rathner’s opinion, countries of the former Eastern Bloc undergoing Westernisation are challenged with “first, the transition to capitalism with all the imponderables of early capitalist accumulation within a few years, and second, the immediate confrontation with globalisation and modernisation with their attendant disruptions” ([4], p. 94). One may assume that the socio-cultural connotations of these disorders in Poland may differ from those observed in Western countries, where they have been observed and studied much longer [46]. Significant differences may include the low percentage of patients diagnosed with EDs in earlier generations, the ethnic coherence of the contemporary Polish population, very low immigration, the high percentage of persons of the Catholic faith, the dynamic process of political transformation and major class-related changes after 1989, as well as other meanings attributed to body weight and shape in the generations raised in communist Poland [46–48]. The dynamic development of the media from 1989 up to the beginning of the 21st century may also be an important factor. During this time, Poland recorded a three-fold increase in the number of press titles to more than 6000 and an increase from two to 80 television stations, and witnessed the dynamic growth of the Internet [49, 50], including the emergence of phenomena related to the *Pro-ana* and *Mia* movements [51].

The aims of this research were to investigate socio-cultural risk factors for ED previously identified in Western societies, now within a Polish context. The study focused on objective measures of the socio-demographic situation of patients and the self-rated importance of socio-cultural context.

## Methods

The presented research was a part of a larger research project entitled “Socio-cultural, familial, and individual factors in anorexia and bulimia nervosa”, which was financed by a scientific grant from the State Committee for Scientific Research (Grant Number: 6 POSE 09021).

### Participants

The study was conducted between 2002 and 2004 on a group of teenage female patients who presented with an ED for the first time to the Child and Adolescent Psychiatry Clinic of the University Hospital in Krakow. The parents of the enrolled patients also participated. Patients and their parents were asked to fill in questionnaires used in the study. EDs diagnoses, based on clinical interview, were made according to DSM-IV criteria [52]. No one refused to participate in the study. Patients with uncertain diagnosis (20 persons), mentally handicapped (4 persons) and being in foster care (10 persons) were not included in the study.

The study included 47 female patients with restrictive anorexia nervosa [ANR], 16 patients with binge/purge anorexia [ANBP], 34 patients with bulimia nervosa (BN) and 19 patients with an eating disorder not otherwise specified [EDNOS]. The study also included an age-matched control group (NOR) of 85 girls (and theirs parents) from Krakow schools. Schools were selected based on a stratified sampling of all the Krakow schools. All chosen schools were public. Voluntary participation in the study was proposed during parent-teachers nights.

### Assessment

The objective socio-demographic situation was quantified using family structure, age, education and employment status of parents. The cultural factors were evaluated using the statements’ form Questionnaire of Socio-Cultural Context (QSCC) designed by M. Pilecki and B. Józefik [53, 54]. The QSCC consists of 62 statements related to the respondent’s views and beliefs on issues that are considered significant cultural risk factors in the development and course of ED, and included statements of Polish specificity (Table **1**). The statements are presented in a random order and responses are made using a six-point Likert scale ranging from 1 (the statement does not apply to me at all) to 6 (the statement describes me very well).

**Table 1:** Items in the Questionnaire of Socio-Cultural Context

1. A person should control his various weaknesses. 2. It is important to the members of my family that we be “up-to-date”. **3. I know many people who have problems with food.** 4. Sex is decidedly a much more enjoyable experience for men than for women. **5. The principles governing my family could be a model for others.** **6. In my social circles, appearance is crucial to a successful social life.** **7. Anorexia and bulimia nervosa are an important issue nowadays.** 8. Currently, fashion sets the rules of conduct in many areas. **9. It can happen that I carry on an internal struggle with myself.** **10. Women in my family do not have an easy life.** 11. We live in times when people are guided more by their own interests than, for example, loyalty and friendship. **12. When I see a thin model, I feel fat and unattractive.** **13. A slim figure may sometimes help more in life than high intelligence.** **14. If someone is not able to control how much and what they eat, they are not able to control anything at all.** 15. Lately, everything is changing so fast, it is difficult to keep up. **16. To be more attractive, I should be skinnier.** **17. My mother has a more difficult life than my father does.** **18. I am jealous of a model’s slim appearance.** 19. Boys can be themselves to a greater degree than girls can. **20. My father pays attention to his figure.** 21. Often, an attractive appearance can only be achieved through hard work on your body. 22. I am sometimes guided in my decisions by advertisements. 23. In life, you should be striving for perfection. **24. Anorexia and bulimia are a problem of my generation.** 25. My father attaches great importance to his external appearance. 26. In today’s times, it is difficult to be yourself in relations with boys. 27. I am religious person. **28. Sometimes, I do not know what is appropriate and what not in relations with others.** **29. I try to eat “healthy food”.** **30. My greatest desire is to know how to control my life.** **31. My mother pays attention to her figure.** 32. I believe that my family is materially better off than other families. **33. Currently, fashionable clothing is tailored so that only thin girls can fit into it.** 34. People who devote themselves to others are fools. **35. If I could get my weight lower or higher by a few kilograms, my female classmates would immediately notice.** **36. A woman should strive to have a profession that allows her to be independent.** **37. Sometimes I succumb to the temptations of my body.** 38. In my social circle, friendliness is what decides your social success. **39. What my family considers delicious food is unhealthy in my opinion.** 40. I believe that observing fasts for religious reasons has deep meaning. **41. True excellence is the ability to have full control over all your feelings.** **42. When I hear about people who have problems with eating, such as anorexia and bulimia, I understand them very well.** 43. Religion is very important in the life of my family. 44. In the world of film and fashion, there are people I would like to resemble. 45. Sometimes it seems to me that there is a large gap between how my parents think and how my grandparents think. 46. It has happened that I have allowed my decisions to be guided by the tastes of well-known models and actresses. 47. Intuition directs us better than logic and planning. 48. Adjusting to today’s times requires a great deal of effort on the part of my family. 49. Healthy competition can be fun. 50. It happens that others are jealous of my good grades. 51. I know at least a few people that are trying to lose weight. 52. Faith does not have great significance for me. **53. The opinion of others is very important to members of my family.** 54. Sons have it easier in Polish families than daughters. **55. I feel that my peers pay attention to how much I eat.** 56. If I am not successful in life, my parents will be disappointed. 57. My mother attaches great importance to her external appearance. 58. Food advertisements are the most difficult to resist. 59. I learn more about how to live from my father than my mother. 60. We will be punished for our sins. **61. My family belongs to the “middle-class”.** **62. If pregnancy were to make my body less attractive, then I would give some thought to whether I want to have any children at all.**	

Items statistically differed between groups are presented in bold

### Statistical analysis

Categorical results are presented as number and percentage. Relationships between two given categorical features were investigated using the chi-square test or Fisher’s exact test in case of insufficient theoretical size. The decomposition of chi-squre test was applied in case of significant results to give a better understanding of the relationship between the two variables. A contingency table which gives rise to a chi-square with p degrees of freedom were decomposed into as many as p smaller tables. Quantitative results, such as the girls’ age and questionnaire responses are presented as mean ± standard deviation, median (lower, Q_1_, and upper, Q_3_, quartile) and minimum and maximum value. Those data were compared across groups (NOR, ANR, ANBP, BN, EDNOS) using the Kruskal-Wallis test due to the lack of normality in case of the age and the overall QSCC scores or due to the ordinal scale of responses of each item. The Shapiro-Wilk test was used to assess normality. In this case the Bonferroni correction was applied to resolve the problem of multiple testing and of finding at least one significant result just due to chance. The significance level was adjusted by the number of tests corresponding with the number of analysed items and the cut-off point was set to be less than .0001.

In any other case *p* value < .05 was considered statistically significant. The analyses were performed using SPSS Statistics v.21 (IBM, New York, USA).

### Ethics

The study was approved by the Bioethics Commission UJ CM No: KBET/26/B/2001.

## Results

The percentage of patients with complete and incomplete families was significantly different across groups (*p* = .013, Fisher’s exact test, Table **2**). The proportion of girls with complete families was higher in the control group than in the other groups. Significant differences were observed between median of patients’ age (*p* = .022, Kruskal-Wallis test, Table **2**). The post-hoc test showed that the ANR group (16.34 (15.0–18.0) years) differed significantly from the BN group (17.53 (17.0–18.0) years) (*p* = .013).

**Table 2:** Socio-demographic characteristics of patient and control groups

		NOR	ANR	ANBP	BN	EDNOS	*p*-value
Number		85	47	16	34	19	
Age (years)	mean ± SD	17.00 ± 1.59	16.34 ± 1.58	16.75 ± 1.29	17.53 ± .96	17.0 ± 1.37	.022
	Q1-Q3	17 (16–18)	16 (15–18)	17 (15.25–18)	17.5 (17–18)	17 (16–18)	
	min-max	13–19	13–19	15–18	15–20	15–19	
	n	85	47	16	34	19	
No. of families with both biological parents		70 (89.7 %)	34 (72.3 %)	12 (75.0 %)	21 (63.3 %)	15 (83.3 %)	.013
n		78	47	16	33	18	
Mother’s age	<35 years	2 (2.4 %)	1 (2.1 %)	1 (6.3 %)	1 (2.9 %)	0 (0 %)	.570
	36–45 years	48 (57.8 %)	29 (61.7 %)	9 (56.3 %)	25 (73.5 %)	14 (73.7 %)	
	46–55 years	31 (37.3 %)	13 (27.7 %)	6 (37.5 %)	8 (23.5 %)	5 (26.3 %)	
	>56 years	2 (2.4 %)	4 (8.5 %)	0 (0 %)	0 (0 %)	0 (0 %)	
	n	83	47	16	34	19	
Father’s age	<35 years	0 (0 %)	0 (0 %)	1 (7.7 %)	1 (4.0 %)	0 (0 %)	.154
	36–45 years	28 (35.0 %)	17 (43.6 %)	3 (23.1 %)	14 (56.0 %)	9 (50.0 %)	
	46–55 years	45 (56.3 %)	20 (51.3 %)	9 (69.2 %)	8 (32.0 %)	9 (50.0 %)	
	>56 years	7 (8.8 %)	2 (5.1 %)	0 (0 %)	2 (8.0 %)	0 (0 %)	
	n	80	39	13	25	18	
Mother’s education level	Primary	14 (16.9 %)	7 (25.9 %)	3 (18.8 %)	8 (25.0 %)	2 (11.1 %)	.740
	Secondary	41 (49.4 %)	16 (36.4 %)	8 (50.0 %)	12 (37.5 %)	10 (55.6 %)	
	Higher	28 (33.7 %)	21 (29.6 %)	5 (31.3 %)	12 (37.5 %)	6 (33.3 %)	
	n	83	44	16	32	18	
Father’s education level	Primary	20 (27.8 %)	10 (27.8 %)	7 (53.8 %)	12 (50.0 %)	6 (35.3 %)	.317
	Secondary	26 (36.1 %)	14 (38.9 %)	2 (15.4 %)	7 (29.2 %)	8 (47.1 %)	
	Higher	26 (36.1 %)	12 (33.3 %)	4 (30.8 %)	5 (20.8 %)	3 (17.6 %)	
	n	72	36	13	24	17	
Place of residence	Village	13 (16.5 %)	13 (31.0 %)	3 (18.8 %)	10 (32.3 %)	4 (26.7 %)	<.001
	Small town	5 (6.3 %)	14 (33.3 %)	9 (56.3 %)	9 (29.0 %)	5 (33.3 %)	
	Large city	61 (77.2 %)	15 (35.7 %)	4 (25.0 %)	12 (38.7 %)	6 (40.0 %)	
	n	79	42	16	31	15	
Mother’s professional circumstance	Employed	69 (84.1 %)	36 (81.8 %)	8 (50.0 %)	21 (65.6 %)	16 (88.9 %)	.031
	Pension	3 (3.7 %)	2 (4.5 %)	3 (18.8 %)	6 (18.8 %)	0 (0 %)	
	Retired	0 (0 %)	2 (4.5 %)	0 (0 %)	0 (0 %)	0 (0 %)	
	Unemployed	7 (8.5 %)	2 (4.5 %)	4 (25.0 %)	4 (12.5 %)	2 (11.1 %)	
	Other	3 (3.7 %)	2 (4.5 %)	1 (6.3 %)	1 (3.1 %)	0 (0 %)	
	n	82	44	16	32	18	
Father’s professional circumstance	Employed	58 (81.7 %)	29 (87.9 %)	8 (72.7 %)	19 (86.4 %)	17 (94.4 %)	.435
	Pension	3 (4.2 %)	2 (6.1 %)	0 (0 %)	1 (4.5 %)	0 (0 %)	
	Retired	2 (2.8 %)	0 (0 %)	0 (0 %)	2 (9.1 %)	0 (0 %)	
	Unemployed	7 (9.9 %)	1 (3.0 %)	3 (27.3 %)	0 (0 %)	1 (5.6 %)	
	Other	1 (1.4 %)	1 (3.0 %)	0 (0 %)	0 (0 %)	0 (0 %)	
	n	71	33	11	22	18	

Quantitative data are presented as mean ± SD, median (interquartile range) and min–max value. Qualitative data as N (%)

*Abbreviations*: *NOR* controls, *ANR* anorexia nervosa restrictive type, *ANBP* anorexia binge/purge type, *BN* bulimia nervosa, *EDNOS* eating disorder not otherwise specified

Maternal and paternal age did not differ significantly between the groups (*p* = .570 and *p* = .154, respectively, Fisher’s exact test, Table **2**). Likewise, maternal and paternal level of education did not differ significantly between groups (*p* = .740 and *p* = .317, respectively, Fisher’s exact test; Table **2**).

The percentage of patients living in villages, small towns and large cities was significantly different across groups (*p* < .001, chi-square test, Table **2**). Most patients in the BN and ANR groups lived in a rural village (ca. 30 %), the most patients in the ANBP groups lived in a small town (ca. 56 %), and the most controls lived in Krakow, a large city (ca. 77 %). The proportion of mothers who were living on a pension, retired or unemployed was significantly different across groups (*p* = .031, Fisher’s exact test; Table **2**). In all groups, the vast majority of mothers worked (81–89 % in the NOR, ANR and EDNOS groups, 66 % in the BN group and 50 % in the ANBP group). The proportion of fathers who were living on a pension, retired or unemployed was not significantly different across groups (*p* = .435, Fisher’s exact test; Table **2**).

29 of the 62 items on the QSCC were able to statistically distinguish between groups. Those items are shown in bold in Table **1**, whereas descriptive statistics of the items with results of post-hoc tests are presented in Table 3.

**Table 3:** Descriptive characteristics of items that differ significantly between groups

Item	NOR	ANR	ANBP	BUL	EDNOS	*p* value*
3	2.96 ± 1.45	3.07 ± 1.60	3.36 ± 1.45	4.03 ± 1.66	3.33 ± 1.68	NOR–BN p = .030
	3 (2–4)	3 (2–4)	3 (2–5)	4 (2.5–6)	3.5 (2–5)	
	1–6	1–6	1–6	1–6	1–6	
5	3.78 ± 1.41	3.17 ± 1.27	2.53 ± 1.25	2.71 ± 1.53	2.72 ± 1.45	NOR–BN p = .005 NOR–ANBP p = .033
	4 (3–5)	3 (2–4)	3 (1–4)	3 (1–4)	3 (1–4)	
	1–6	1–6	1–4	1–6	1–6	
6	3.14 ± 1.42	2.74 ± 1.35	3.67 ± 1.29	3.61 ± 1.45	3.94 ± 1.52	NOR–BN p = .005 NOR–ANBP p = .033
	3 (2–4)	2.5 (2–4)	4 (3–5)	3 (2–5)	4 (3–5)	
	1–6	1–6	1–6	1–6	1–6	
7	4.49 ± 1.52	5.30 ± 1.04	5.0 ± 1.25	5.45 ± 0.77	5.28 ± 0.75	NOR–ANR p = .010 NOR–BN p = .011
	5 (3–6)	6 (5–6)	5 (5–6)	6 (5–6)	5 (5–6)	
	1–6	2–6	1–6	3–6	4–6	
9	4.07 ± 1.52	4.72 ± 1.22	5.33 ± 1.05	5.45 ± 1.09	4.94 ± 1.47	NOR–ANBP p = .010 NOR–BN p < .001
	4 (3–5)	5 (4–6)	6 (5–6)	6 (5–6)	5.5 (4–6)	
	1–6	2–6	3–6	1–6	1–6	
10	2.58 ± 1.53	3.17 ± 1.56	3.53 ± 1.46	4.26 ± 1.69	3.67 ± 1.24	NOR–BN p < .001
	2 (1–4)	3 (2–4)	4 (3–5)	5 (3–6)	3.5 (3–4.25)	
	1–6	1–6	1–6	1–6	2–6	
12	3.38 ± 1.64	3.19 ± 1.76	5.07 ± 1.39	4.84 ± 1.44	4.67 ± 1.50	ANR–BN p = .001 ANR–ANBP p = .002 ANR-EDNOS p = .026 NOR–BN p = .001 NOR–ANBP p = .004 NOR-EDNOS p = .043
	3 (2–5)	3 (1–5)	6 (5–6)	5 (4–6)	5 (4–6)	
	1–6	1–6	2–6	1–6	1–6	
13	3.77 ± 1.40	3.28 ± 1.52	5.07 ± 1.39	4.03 ± 1.56	4.44 ± 1.58	ANR-EDNOS p = .048
	4 (3–5)	3 (2–4)	6 (5–6)	4 (3–5)	5 (3.75–6)	
	1–6	1–6	2–6	1–6	1–6	
14	2.78 ± 1.19	2.58 ± 1.22	3.73 ± 1.67	4.0 ± 1.59	4.28 ± 1.41	ANR–BN p = .001 ANR–EDNOS p = .001 NOR–BN p = .002 NOR–EDNOS p = .002
	3 (2–4)	3 (2–3)	3 (2–5)	4 (3–5)	4 (3–6)	
	1–6	1–5	1–6	1–6	1–6	
16	3.46 ± 1.75	2.50 ± 1.49	4.07 ± 1.79	4.74 ± 1.46	4.50 ± 1.79	ANR–NOR p = .049 ANR–ANBP p = .037 ANR–EDNOS p = .001 ANR–BN p < .001 NOR–BN p = .010
	3 (2–5)	2 (1–4)	4 (2–6)	5 (4–6)	5 (3.5–6)	
	1–6	1–6	1–6	1–6	1–6	
17	2.91 ± 1.69	3.10 ± 1.99	4.13 ± 1.41	4.29 ± 1.72	3.82 ± 1.85	NOR–BN p = .005
	3 (1–4)	3 (1–5)	4 (3–6)	4 (3–6)	4 (2–5.5)	
	1–6	1–6	2–6	1–6	1–6	
18	3.42 ± 1.72	3.09 ± 1.57	4.73 ± 1.53	4.77 ± 1.67	4.72 ± 1.74	ANR–ANBP p = .017 ANR–BN p < .001 ANR-EDNOS p = .008 NOR–BN p = .002 NOR-EDNOS p = .043
	3 (2–5)	3 (1–4)	5 (4–6)	6 (4–6)	5.5 (3–6)	
	1–6	1–6	1–6	1–6	1–6	
20	2.78 ± 1.45	2.14 ± 1.52	2.0 ± 1.0	1.77 ± 1.04	1.94 ± 1.09	NOR–BN p = .006 NOR–ANR p = .044
	3 (2–4)	1 (1–3)	2 (1–3)	1 (1–2.25)	2 (1–3)	
	1–6	1–6	1–4	1–4	1–4	
24	3.961.45	4.60 ± 1.50	4.80 ± 1.37	5.13 ± 1.31	4.83 ± 1.54	NOR–BN p = .001
	4 (3–5)	5 (3–6)	5 (4–6)	6 (5–6)	5.5 (4–6)	
	1–6	1–6	1–6	1–6	1–6	
28	3.13 ± 1.39	4.09 ± 1.56	3.47 ± 1.13	3.65 ± 1.52	3.44 ± 1.50	NOR–ANR p = .009
	3 (2–4)	4 (3–6)	4 (2–4)	4 (2–5)	3.5 (2–4.25)	
	1–6	1–6	2–5	1–6	1–6	
29	3.62 ± 1.23	5.0 ± 1.16	5.13 ± 0.99	4.39 ± 1.26	4.89 ± 1.41	NOR–EDNOS p = .002 NOR–ANR p < .001 NOR–ANBP p = .001
	4 (3–4.5)	5 (4–6)	5 (4–6)	4 (3–6)	5.5 (4–6)	
	1–6	1–6	3–6	2–6	1–6	
30	3.90 ± 1.31	4.12 ± 1.55	5.33 ± 0.90	5.32 ± 0.95	4.67 ± 1.41	NOR–BN p < .001 NOR–ANBP p = .002 ANR–BN p = .003 ANR–ANBP p = .039
	4 (3–5)	4 (3–6)	6 (5–6)	6 (5–6)	5 (4–6)	
	1–6	1–6	3–6	3–6	1–6	
31	3.59 ± 1.35	2.98 ± 1.42	2.93 ± 1.34	3.16 ± 1.53	2.39 ± 1.46	NOR–EDNOS p = .014
	4 (2.5–5)	3 (2–4)	3 (2–3)	3 (2–4)	2 (1–4)	
	1–6	1–6	1–6	1–6	1–5	
33	3.67 ± 1.56	3.33 ± 1.60	4.33 ± 1.80	4.39 ± 1.61	4.61 ± 1.46	ANR-EDNOS p = .048
	3 (3–5)	3 (2–5)	5 (3–6)	5 (3–6)	5 (4–6)	
	1–6	1–6	1–6	1–6	1–5	
35	3.49 ± 1.67	4.16 ± 1.60	4.73 ± 1.39	4.57 ± 1.52	4.33 ± 1.78	NOR–BN p = .030
	3 (2–5)	4 (3–6)	5 (4–6)	5 (3–6)	5 (2.75–6)	
	1–6	1–6	2–6	1–6	1–6	
36	4.51 ± 1.19	4.86 ± 1.22	5.33 ± 0.90	5.33 ± 0.96	5.11 ± 1.13	NOR–BN p = .005
	5 (4–5)	5 (4–6)	6 (5–6)	6 (5–6)	5.5 (4.75–6)	
	1–6	1–6	3–6	2–6	3–6	
37	3.19 ± 1.46	3.40 ± 1.28	3.93 ± 1.39	4.17 ± 1.20	3.78 ± 1.48	NOR–BN p = .026
	3 (2–4)	4 (2–4)	4 (3–5)	4 (3–5)	4 (2.75–5)	
	1–6	1–6	1–6	2–6	1–6	
39	2.73 ± 1.26	3.86 ± 1.34	4.80 ± 1.27	3.87 ± 1.57	4.50 ± 1.25	NOR–BN p = .006 NOR–ANR p = .001 NOR–EDNOS p < .001 NOR–ANBP p < .001
	3 (2–3)	4 (3–5)	5 (4–6)	4 (2–5)	4.5 (3.75–6)	
	1–6	1–6	2–6	1–6	2–6	
41	3.78 ± 1.26	4.02 ± 1.35	4.27 ± 1.62	4.73 ± 1.39	4.50 ± 1.43	NOR–BN p = .009
	4 (3–5)	4 (3–5)	5 (3–6)	5 (3–6)	5 (3.75–5.25)	
	1–6	1–6	1–6	1–6	1–6	
42	2.88 ± 1.33	5.02 ± 1.24	5.47 ± 1.13	5.47 ± 1.04	5.06 ± 1.39	NOR–EDNOS p < .001 NOR–ANR p < .001 NOR–ANBP p < .001 NOR–BN p < .001
	3 (2–4)	6 (4–6)	6 (5–6)	6 (5–6)	6 (4.75–6)	
	1–6	2–6	2–6	2–6	2–6	
53	3.11 ± 1.26	3.33 ± 1.24	3.80 ± 1.08	4.17 ± 1.28	3.67 ± 1.46	NOR–BN p = .004
	3 (2–4)	3 (2–4)	4 (3–5)	4 (3–5)	4 (2–5)	
	1–6	1–6	2–6	2–6	1–6	
55	2.38 ± 1.52	4.05 ± 1.55	4.53 ± 1.46	4.13 ± 1.66	4.06 ± 1.70	NOR–EDNOS p = .003 NOR–ANR p < .001 NOR–BN p < .001 NOR–ANBP p < .001
	2 (1–3.5)	4 (3–5)	5 (4–6)	5 (3–5.25)	3.5 (3–6)	
	1–6	1–6	2–6	1–6	1–6	
61	3.99 ± 1.48	4.78 ± 1.28	4.0 ± 1.62	4.77 ± 1.04	4.94 ± 0.94	NOR–ANR p =,027
	4 (3–5)	5 (4–6)	4 (3–5.25)	5 (4–6)	5 (4.75–6)	
	1–6	1–6	1–6	3–6	3–6	
62	1.78 ± 1.22	2.21 ± 1.47	2.47 ± 1.51	3.13 ± 1.85	2.56 ± 1.62	NOR–BN p = .002
	1 (1–2)	2 (1–3)	2 (1–4)	3 (1–5)	2 (1–4)	
	1–6	1–6	1–6	1–6	1–4	

Quantitative data are presented as mean ± SD, median (interquartile range) and min–max value

*Abbreviations*: *NOR* controls, *ANR* anorexia nervosa, *ANBP* anorexia binge/purge type, *BN* bulimia nervosa, *EDNOS* eating disorder not otherwise specified

*p-value obtained from the post-hoc tests. The numbers presented in the table indicate the amount of answers received. Information regarding the lack of data was omitted

## Discussion

The aim of this study was to assess the relationship between the clinical characteristics of EDs and socio-cultural factors in Poland. We considered factors that objectively describe the socio-economic status of patients as well as the importance that patients attached to various culture aspects described as important risk factors for the development and course of EDs. This is the first exploratory study of socio-cultural aspects of Eds in Poland and one of the firsts in Eastern Europe.

Objective descriptors of social affiliation did not distinguish girls with EDs from the control group. An important source of potential bias is the recruitment of the control group from female high school students in Krakow. The girls in the EDs groups more frequently came from villages and small towns. This may mean that living in smaller populated areas, for some reason, may be connected with the significant cultural risk of developing eating disorders in Poland. Other possibility is that girls from larger cities, especially Krakow, have access to private healthcare. Althought interpreting this result is not simple. It does not necessarily define a different membership to social groups of people living in various areas. After the year 1989, living in the countryside or in a smaller town may be a social advancement and moving to a detached house build outside large urban agglomerations may be interpreted as such. There was a significant difference in the professional activity of mothers across different diagnoses. The proportion of mothers that worked was lower in the BN and ANBP groups than in the NOR and ANR groups. The most common reason for the lack of professional activity was a pension. The comparatively higher percentage of single parent families, especially in the patients with BN, may indicate a significant level of socio-economic crisis in this families. An objective analysis of the family situation does not allow clear conclusions regarding social class and the occurrence of EDs. However, our patients tended to come from a particular group. According to Central Statistical Office of Poland in 2002, 9.3 % of men and 10.4 % of women in Poland had higher education, 30.1 % of men and 16.9 % of women had basic vocational training, and 28 % of men and 31.4 % of women had primary education [55]. Compared to these sociological data, the education level of the parents of our patients was higher than average, with an over-representation of parents having a high level of education and an under-representation of those with vocational and primary education. This confirms data from research from other countries [11].

Only a subset of the QSCC variables significantly distinguished patients with EDs from the control group. The NOR group was distinguishable from the BN group by the greatest number of items (23), whereas the ANR group was distinguishable from NOR by 7 items, and ANBP and EDNOS by 9.

A negative assessment of the dietary rules prevailing in the family house in comparison with control group (item 39 on the QSCC) was common to all the girls with EDs diagnosis. For the BN and ANR groups, this was associated with the belief that the father cared very little about his figure (item 20). In the EDNOS group, a similar relationship was observed with respect to mothers (item 3l). One possible interpretation is that the patients came from homes where diet and appearance were not important. Given Poland’s history, it does not mean that the patients came from families of a lower social class. The parents of the patients grew up in communist Poland. The early 1980s was a time of deep economic crisis in Poland, with rationing of food and a chronic shortage of supplies. Therefore, the relationship that people of this generation have with food has been subject to the same processes as any society emerging from poverty and shortage, where obesity, once a signifier of high economic status, becomes a symbol of poverty and backwardness. This change is slower in men than in women [56].

A focus on eating and appearance may also be the result of patient attitudes that are not shared by their parents. In the BN group, in particular, the meaning ascribed to thinness may be an expression of an attempt to distinguish oneself from one’s parents and not follow them. In addition, the response of the patients may not be an objective perception of parental attitudes, but rather an expression of strict standards and rules these girls apply to their diets and their personal hygiene. No interviews were conducted during the study to determine the real attitudes of parents. However, the studied mothers achieved very low scores in the EAT26 test, indicating an absence of problems with eating and their relationship to their own bodies, although this did include a lack of interest in dietary standards and principles [47]. In particular, in the BN group, a negative evaluation of dietary standards in the family was associated with an equally negative assessment of family relations and behaviour, including the position of women and mothers in the family (items 53, 39, 10, and 17). In the ANR group, there was an association with the belief in the family’s membership that they are in the middle class (item 61). Two additional items characteristic of all patient groups concerned peer groups and included understanding other people suffering from similar problems (item 42) and a belief that peers pay attention to how much the patients eat (item 55). In the BN group, patients knew other people who have problems with food (item 3) and felt that their peers would notice even small changes in their weight (item 35). This confirms previous observations of the importance of peer relationships to problems associated with the relationship to one’s own body and with eating, including in their pathological forms [28, 29, 39]. The response to item 55 (I feel that my peers pay attention to how much I eat) may be an expression of a real interest in ways of losing weight (ANR and ANBP groups) or concern for a gorging-and-vomiting classmate (BN and ANBP groups). The perception of EDs as a major problem of modern teenagers (item 24 for the BN group and item 7 for the ANR and BN groups) was also able to distinguish patients with ED from controls. On several items, the ANBP group behaved in a manner similar to the BN group, supporting the intermediate character of this group [57]. The variation in the responses of the EDNOS group points to internal inconsistencies associated with the inclusion of patients diagnosed with different subclinical states [58].

There were no statistically significant differences between groups for any item related to the importance of advertising or religious context. In the case of advertising, this result is different from majority of results within the Western cultural sphere [30], and this difference may be due to the fact that, at the time of the study, the media market in Poland was less developed than in Western countries.

Would the results suggest that cultural context is relevant for BN but not for ANR? For Hilde Bruch [59], a distorted body image is central to the symptomology of ED, and the more body image is distorted, the more pronounced are the symptoms. What may be significant here are the relationships identified by items 12, 16 and 18, which indicate the irrelevance of thin pressure in the ANR group. It is possible that thin pressure was relevant before the manifestation of ED, but then lost its relevance following significant weight loss. This is not however observed in ANBP group. This indicates an interpretive trap, which similar studies may fall, not taking into account the type of eating disorders or anorexia nervosa.

These results may simply show that girls with restrictive type anorexia came from families without relationship problems [60, 61] and did not deviate significantly from the standard self-image [57]. However, EDs patients’ images of family relationships are not generally as optimistic when one asks their parents [61]. Symptom denial is an important aspect of ED, and may affect not only the patient’s somatic state, but also other relevant aspects of the patient’s self-image and perception of relation to the world [62, 63]. This brings the credibility of any self-assessment test performed by girls suffering from ANR into question, including self-assessment tests of cultural issues.

Our study has some important limitations. Data were collected only from one university hospital. The socio-cultural factors in samples from other clinical centres, community mental centres or private practises located in different areas of Poland could have been different. The recruitment of the control group based on parents volunteering and limited to girls from public schools within Krakow only can influence the results too.

It should be noticed that QSCC is a quite new tool and needs further exploration. The previous study [54] presents the results of the factor analysis of QSCC based on 614 schoolgirls from Krakow, aged 15–19 (mean: 16,83 years; SD: 1,04 years). The results of Cronbach’s alphas (≥.7) and loadings (≥.35) of created 7 subscales were satisfactory and reflect 50.4 % of the total variability, but there were a lot of doubts about applying these subscales among girls with eating disorders. According to this the current paper presents the results based only on each item separately.

## Conclusions

The results of this study indicate the importance of both objective social data and subjective cultural factors in EDs in the group studied. However, whether the cultural context is a true risk factor, proxy risk factor, mediating factor, potentiating factor, causal factor, maintenance factor or — in some dimensions — a protective factor cannot be determined from this type of analysis. A more clear-cut understanding of the meaning of the examined factors requires a longitudinal study that allows the emergence of EDs to be associated with an earlier interaction of variables (e.g., attitude towards one’s body and eating habits) in persons without an initially occurring pathology.
